# Clinical profile and treatment of infantile spasms using vigabatrin and ACTH - a developing country perspective

**DOI:** 10.1186/1471-2431-10-1

**Published:** 2010-01-15

**Authors:** Shahnaz Ibrahim, Shamshad Gulab, Sidra Ishaque, Taimur Saleem

**Affiliations:** 1 Department of Pediatrics and Child Health, Aga Khan University, Stadium Road, Karachi 74800, Pakistan; 2 Medical College, The Aga Khan University, Stadium Road, Karachi 74800, Pakistan

## Abstract

**Background:**

Infantile spasms represent a serious epileptic syndrome that occurs in the early infantile age. ACTH and Vigabatrin are actively investigated drugs in its treatment. This study describes the comparison of their efficacy in a large series of patients with infantile spasms from Pakistan.

**Methods:**

All patients with infantile spasms who presented to Aga Khan University Hospital, Karachi, Pakistan from January, 2006 to April, 2008 were included in this study. Inclusion criteria were clinical symptoms of infantile spasms, hypsarrythmia or modified hyparrythmia on electroencephalography, at least six months of follow-up period and receipt of any of the two drugs mentioned above. The type of drug distribution was random according to the availability, cost and ease of administration.

**Results:**

Fifty six cases fulfilled the inclusion criteria. 62.5% were males. Mean age at onset of seizures was 5 ± 1.4 months. Fifty two (92.8%) patients demonstrated hypsarrythmia on electroencephalography. 64.3% cases were identified as symptomatic while 19.6% were cryptogenic and 16.1% were idiopathic. Eighteen patients received ACTH while 38 patients received Vigabatrin as first line therapy. Initial response to first line therapy was similar (50% for ACTH and 55.3% for Vigabatrin). Overall, the symptomatic and idiopathic groups responded better to Vigabatrin. The relapse rate was higher for ACTH as compared to Vigabatrin (55.5% vs. 33.3%) when considering the first line therapy. Four patients evolved to Lennox-Gastaut variant; all of these patients had initially received Vigabatrin and then ACTH.

**Conclusion:**

Vigabatrin and ACTH showed no significant difference in the initial treatment of infantile spasms. However, patients receiving ACTH were 1.2 times more likely to relapse as compared to the patients receiving Vigabatrin when considering monotherapy. We suggest that Vigabatrin should be the initial drug of choice in patients presenting with infantile spasms. However, larger studies from developing countries are required to validate the therapeutic trends observed in this study.

## Background

Infantile spasms represent a unique and age-specific epilepsy disorder usually presenting in early infancy. If left untreated, infantile spasms are associated with devastating neurological results. This severe epilepsy syndrome is often considered an age-dependent expression of a damaged brain [1].

The incidence of infantile spasms ranges from 0.25 - 0.6 per 1000 live births [2]. Electroencephalography (EEG) characteristically demonstrates the hypsarrythmic pattern. In addition, there is neurodevelopment regression [3]. Infantile spasm may be seen either alone or in the constellation of West Syndrome. West syndromee consists of the triad of infantile spasms, an interictal EEG pattern (hypsarrhythmia or similar pattern), and mental retardation. The key features to diagnosing infantile spasms are patient history, age, characteristic semiology of seizures, and distinctive EEG pattern [3].

These seizures typically present with a triad of lightning (involving the entire body), nodding (convulsions of the throat and neck flexor muscles) and Salaam or jackknife attacks (rapid bending of the head and torso forward and simultaneous raising and bending of the arms) [1,4]. Infantile spasms are distinct from myoclonic and tonic seizures. They are characterized by a contraction phase followed by a more tonic phase. The contraction phase may be a flexor spasm, extensor spasm, or a combination of both and these could be symmetrical or asymmetrical.

The frequent onset of infantile spasms in infancy suggests that an immature central nervous system may be important in its pathogenesis. The brain-adrenal axis may also be involved. It is proposed that the effect of different stressors in the immature brain produces an abnormally excessive secretion of corticotropin-releasing hormone (CRH), causing spasms. The clinical response to adrenocorticotropic hormone (ACTH) and glucocorticoids can be therefore be explained by the suppression of CRH production [1].

The treatment of infantile spasms has been both a conundrum and a challenge for the pediatric neurologist as the entity appears to be resistant to many conventional antiepileptic drugs. Agents that have been employed in the treatment of infantile spasms include benzodiazepams (especially nitrazepam), sodium valproate, vigabatrin (VGB), corticosteroids, ACTH, a ketogenic diet, vitamin B6, intravenous gammaglobulin, a benzodiazepam-carbamazepine cocktail, topiramate and zonisamide [2,5].

Currently ACTH and vigabatrin are the actively investigated drugs with some evidence of efficacy in the treatment of infantile spasms. However, there is little consensus with regards to the definitive dose, efficacy or duration of treatment of these agents in comparison to each other [3]. Studies on experience with ACTH and Vigabatrin from Pakistan and neighboring regions are lacking. To date, there has been only one study from Pakistan reporting the use of ACTH and prednisolone [6]. There has been no study exploring the efficacy of Vigabatrin in Pakistan. Therefore, this study was undertaken to provide an important perspective on the treatment of infantile spasms in the setting of a developing country like Pakistan.

## Methods

### Study setting

The Aga Khan University Hospital (AKUH) is a tertiary level teaching facility in the private sector in Karachi, Pakistan. It has approximately 550 beds and provides services to over 38,000 hospitalized patients and to over 500,000 outpatients annually [7]. Patients belonging to all socioeconomic classes and from all over Karachi as well as Pakistan visit to AKUH for treatment.

### Study design

This study included 56 patients with infantile spasms who presented to AKUH Consulting Clinic from January, 2006 to April, 2008. Patient records were reviewed and data was collected on structured proformas especially prepared for this purpose.

### Inclusion and exclusion criteria

Inclusion criteria included the following:

1. Clinical symptomatology of infantile spasms as elicited by thorough physical examination and detailed history

2. Accompanying EEG findings at initial EEG (hypsarrythmia or similar pattern on EEG with almost continuous high voltage multifocal spike and wave discharge and a disorganized/chaotic background)

3. At least six months of follow-up from the time of initial diagnosis

4. Receipt of any of the two drugs mentioned above. The type of drug administration was according to the availability, cost and ease of administration.

Exclusion criteria used for this study were:

1. All children who were diagnosed to have tuberous sclerosis were excluded since there is enough evidence in literature of the advantage of Vigabatrin over steroids in cases of infantile spasms with tuberous sclerosis.

2. Children who had already received any of the investigational drugs prior to their first encounter were also excluded.

### Working definitions

#### a. Response to therapy

Cases were considered as free from infantile spasms either on the basis of clinical resolution of spasms or on the basis of both clinical resolution of spasms and EEG resolution of hypsarrythmia. A follow-up EEG couldn't be performed in all cases unfortunately due to financial constraints cited by some parents. Partial response was considered when the number of spasms was reduced by at least ≥ 50% as compared to the baseline spasms at initial presentation before the administration of any drug. Patients were categorized as having no response when there was no change in the number of spasms even after a period of 6 weeks of adequate therapy.

#### b. Categories based on etiology

All cases were grouped into three categories. Those cases in which infantile spasms occurred without any identifiable cause, other neurological signs or symptoms were considered as idiopathic. Patients who were suspected of being symptomatic but for whom an underlying structural or biochemical cause could not be identified were classified as cryptogenic. Cases were considered as symptomatic when the infantile spasms resulted from an identifiable cause and often where neurologic features or unequivocal developmental delay preceded the onset of symptoms [8].

#### c. Dosages of medications

The starting dose of Vigabatrin varied from 12.5 to 25 mg/kg/day in all the cases while the maximum dosage of Vigabatrin during the course of treatment was 150 mg/kg/day. Increments were done every 3-5 days depending on the tolerance of the patient to the drugs administered. We must mention here that some children became extremely lethargic and drowsy after receipt of the medication; therefore the increments were considerably slower in such children depending on their clinical state. The maximum dosage of Vigabtrin that was given to all cases depended on the response observed.

For ACTH, the typical dosage was 40 International Units (IU). However, in 2 children the dosages were increased to a maximum of 80 units as their initial response was considerably slower.

The children who showed a partial or no response to the initial therapy after the maintenance doses of either of the two medications for adequate time duration of 6 weeks were then put on a second drug. For example, patients who were initially on Vigabatrin but failed to show adequate response were put on ACTH. Similarly, patients who were initially on ACTH but failed to show adequate response were put on Vigabatrin. Patients who showed response to the initial drug were maintained on that therapeutic agent.

### Data management and statistical analysis

The data was coded, entered and analyzed using Windows SPSS version 16.0. In descriptive analysis, the mean and standard deviations of the continuous variables and percentages of categorical variables were computed. Associations were assessed using chi-square test and Fisher's exact test where appropriate. A p-value of < 0.05 was considered statistically significant, unless otherwise specified. Variables with a significant p-value were further subjected to multiple logistic regression analysis to ascertain independent predictors of infantile spasm free final outcome of treatment.

### Ethical considerations

Being a retrospective study, the study protocol was granted exemption from review as per guidelines of the Ethical Review Committee (ERC) of the Department of Pediatrics at Aga Khan University Hospital (AKUH), Karachi. Informed consent was not needed owing to the anonymous presentation of the patient data as per guidelines of the ERC at AKUH.

## Results

Fifty six children with infantile spasms were administered either Vigabatrin or ACTH from 2006-2008. Apart from these 56 patients, an additional two patients had been given prednisolone as initial treatment for infantile spasms. Although they showed complete resolution of the infantile spasms, they were not included as part of primary analysis due to insufficient numbers.

### Clinical profile of patients

#### a. Baseline characteristics

The baseline characteristics of our sample are presented in Table 1. The majority of the patients were male (35/56, 62.5%). Twelve patients (21.4%) were small for gestational age with a birth weight of less than 2.5 kg. A family history of abortions and stillbirths was present in 3 patients (5.3%). A history of neonatal deaths in the family was found in 4 cases (7.1%); however, the definitive cause of death was not known. Three patients (5.3%) had a family member with mental retardation and developmental delay. A family history of infantile spasms was not present in any child. Antenatally, mothers of 3 patients had a history of oligohydramnios during gestation, 2 had a history of typhoid fever (blood culture proven) in the first trimester, and 2 had a history of chorioamnionitis (clinical diagnosis) in the third trimester. Two mothers also had a history of self-resolving heaving bleeding in the first trimester. On presentation 37/56 (66%) children had a fronto-occipital circumference (FOC) less than 5^th ^centile. Four children (7.1%) had brachycephaly on examination. All the patients received an initial EEG. Fifty two (92.8%) patients exhibited the classic hypsarrythmic pattern while a similar pattern such as modified hypsarrythmia was less commonly seen (7.2%). Of the 58 cases of infantile spasms, forty two (75%) patients had not received any prior anti-epileptic medications. The remaining had been treated as non-specific seizure disorder before presenting to our hospital.

**Table 1:** Baseline characteristics of patients presenting with infantile spasms

Characteristics of patients with infantile spasms	n = 56 (%)
Age of onset of seizures in months (Mean ± SD)	5 ± 1.4
Age at diagnosis of infantile spasms in months (Mean ± SD)	6.5 ± 2.3
Lag interval between onset and diagnosis in months (Mean ± SD)	1.4 ± 1.3
Gender (male)	35 (62.5%)
Consanguinity of parents	19 (33.9%)
Seizures per day at onset (Mean ± SD)	62 ± 5.1
Clusters per day at onset (Mean ± SD)	21 ± 4.2
Type of spasms	
-Flexion	23 (41.1%)
-Extension	6 (10.7%)
-Mixed	15 (26.8%)
History of neonatal seizures	15 (26.8%)
History of stay in neonatal intensive care unit	14 (25%)
History of premature birth (< 37 weeks gestation)	14 (25%)
History of neonatal sepsis/meningitis	10 (17.8%)
History of developmental delay prior to onset of symptoms	41 (73.2%)

#### b. Etiology of infantile spasms

In our patient population, 36 (64.3%) of the cases of infantile spasms were symptomatic while 11 (19.6%) were cryptogenic and the remaining 9 cases were idiopathic (16.1%). The criteria used for classifying the cases into symptomatic, cryptogenic and idiopathic types have been mentioned in detail in the Methods section above. Among the symptomatic cases, cerebral palsy secondary to birth asphyxia was identified as the leading cause of infantile spasms (Table 2). It was seen that almost 51.8% (29/56) patients had a history of delayed cry at birth. As per parental recall, the mean time for cry after birth was 7.5 ± 3.5 minutes. APGAR scores were not available in most of the cases since the deliveries since the deliveries had been performed outside our hospital.

**Table 2:** Etiology of Symptomatic Infantile Spasms

Etiology of symptomatic cases of infantile spasms	% (n = 36)
Birth asphyxia	69.4
Complication of pre-maturity	11.1
Post maturity (> 41 weeks gestation)	2.8
Aicardi Syndrome	2.8
Bilirubin encephalopathy (kernicterus)	2.8
Inborn error of metabolism ^	5.5
Post meningitic sequalae	2.8

^ Urea cycle defect and Glutaric Acid deficiency were identified

### Treatment and outcomes

#### a. Initial therapy of infantile spasms

All patients were given one medication at any given time. Out of 56 patients, 18 patients (32.1%) received ACTH while 38 patients (67.9%) received Vigabatrin as initial therapy. The results of initial therapy are presented in Table 3. Initially, 9 out of 18 (50%) patients completely responded to ACTH monotherapy while 21 out of 38 (55.3%) patients completely responded to Vigabatrin monotherapy initially.

**Table 3:** Response of patients to initial therapy

Patient Response to Initial Therapy (p = 0.696)	ACTH n = 18 (%)	Vigabatrin n = 38 (%)
-Complete cessation	9 (50)	21 (55.3)
-Partial response	6 (33.3)	10 (26.3)
-No response	3 (16.7)	7 (18.4)

#### b. Switch to second drug

As mentioned above, 50% and 44.7% patients initially on ACTH and Vigabatrin therapy didn't show adequate response to therapy even after 6 weeks duration. These were patients who showed either partial or no response to the initial therapy. They were then put on a second and different drug. Patients initially on ACTH were now put on Vigabatrin and vice versa. The response of patients to the second drug is shown in table 4.

**Table 4:** Response of patients to second drug that was administered after 6 weeks in case of partial or no response to first drug

Patient Response to Second Drug (p = 0. 334)	ACTH ^ n = 17 (%)	Vigabatrin ^^ n = 9 (%)
-Complete cessation	5 (29.4)	2 (22.2)
-Partial response	5 (29.4)	5 (55.6)
-No response	7 (41.2)	2 (22.2)

^ Patients who had initially received Vigabatrin and shown partial or no response to it

^^ Patients who had initially received ACTH and shown partial or no response to it.

#### c. Relapse

Even among patients who had shown complete resolution of seizures after therapy, some experienced relapse. Fifty percent patients initially put on ACTH showed full response. However, 5 out of these 9 patients (55.6%) relapsed. Similarly, 21 patients had shown full response to Vigabatrin initially. However, 7 out of these 21 patients (33.3%) relapsed. The relapse rate was higher for ACTH as compared to Vigabatrin (55.5% against 33.3%) when considering the initial monotherapy.

The average time for relapse and complete cessation of seizures also yielded interesting findings. It was observed that the patients started on ACTH had earlier relapses and delayed recovery (both clinically and based on EEG findings), as opposed to those started on Vigabatrin therapy. These time durations were 2 weeks for the former and 2 months for the later. Those who were switched to Vigabatrin after prior ACTH therapy relapsed after 6 months on average as opposed to those who were switched to ACTH therapy after prior Vigabatrin therapy, who relapsed after an average time of 2 months. (Table 5) Patients receiving ACTH were 1.2 times more likely to relapse as compared to the patients receiving Vigabatrin when considering monotherapy.

**Table 5:** Final outcome at last follow-up

Therapy	Seizure free till last follow-up	Continued to have seizures	Ave. relapse time	Ave. recovery time ^	Evolved to Lenox Gestaut variant*
ACTH monotherapy (n = 4) ^^	4	0	2 wks	2 months	0
Vigabatrin monotherapy (n = 14) ^^	14	0	2 months	1 month	0
ACTH followed by Vigabatrin (n = 14)	1	13	6 months	3 months	0
Vigabatrin followed by ACTH (n = 24)	2	18	2 months	6 months	4

^ Average time to complete recovery, if occurred

^^ Remained on monotherapy till the last follow-up and didn't receive any second drug

* Evolution by 2 years

#### d. Final outcome of patients

At the time of last follow-up, the cumulative outcome was based either on EEG findings, and/or clinical signs and seizure activity. (Table 5) Only four of the patients initially on ACTH and 14 of the patients initially on Vigabatrin remained seizure free at last follow-up and had not experienced any relapse. Among those who were started on the second drug after incomplete or no response to the first drug, 93% of those on ACTH and 73% of those on Vigabatrin therapy continued to have seizures. It was observed that 4 patients out of the 24 patients who had received ACTH as the second drug evolved to Lennox-Gastaut variant.

The association of the outcome at the last follow-up was significant for the type of treatment received (p < 0.001), the etiology of infantile spasms (p < 0.001) and the age at diagnosis of infantile spasms (p = 0.04). The association of final outcome with age at onset seizures was not significant (p = 0.869). For outcome at last follow-up in terms of clinical and electroencephalographic resolution of seizures, the following variables were subjected to multiple logistic regression analysis: type of treatment received, etiology of infantile spasms and age at diagnosis. (Table 6) It was seen that response to first line treatment, etiology other than symptomatic and younger age at diagnosis are factors associated with resolution of seizures.

**Table 6:** Multiple logistic regression analysis showing predictors for final outcome of infantile spasms in terms of resolution of seizures

Variables	Resolution of Seizures Adjusted OR *	(EEG and/or clinical) 95% CI **
**Type of therapy (p < 0.001)**		
Receipt of second drug	1	-
ACTH monotherapy	4.2	0.6 - 6.8
Vigabatrin monotherapy	4.6	1.1 - 7.2
**Etiology (p < 0.001)**		
Symptomatic	1	-
Cryptogenic	1.2	0.4 - 2.1
Idiopathic	4.4	1.6 - 6.9
**Age at Diagnosis (p = 0.04)**		
> 12 months	1	-
6 - 12 months	1.2	0.8 - 1.5
< 6 months	1.3	0.6 - 2.1

* OR = Odds Ratio

** CI = Confidence Interval

#### e. Association of response to first line therapy with etiology

Considering the seizure outcome with respect to the etiology, it was seen that 30% of all the patients in the symptomatic group responded to monotherapy. Sixty percent of this response was due to Vigabatrin while 34% was due to ACTH. In the cryptogenic group, 36% patients responded to first line therapy. Distribution of this response to first line therapy was 55% and 45% due to Vigabatrin and ACTH respectively. In the idiopathic group, almost 50% patients responded to first line therapy. Vigabatrin accounted for all the responsive cases of idiopathic group with regards to first line therapy.

#### f. Side effects of therapy

Comparing the side effect profile of the drugs, 6 patients developed ACTH induced hypertension while 1 patient developed Vigabatrin induced rash. One patient developed visual field defects secondary to Vigabatrin. However, many patients did not get a complete and detailed eye examination done (including visually evoked potentials and other studies) after being put on Vigabatrin therapy due to non-availability of retinograms at our institution.

#### g. Mortality

A total of 4 patients expired in this study; 2 were receiving vigabatrin at the time of death and their death was attributed to aspiration pneumonia. The remaining 2 were on ACTH therapy and their death was attributed to septicemia. All 4 cases were of symptomatic category of infantile spasms.

## Discussion

Infantile spasms were first described 160 yeas ago in a letter that Dr West wrote to Lancet [9]. Since then atleast 30 drugs have been investigated in the treatment of infantile spasms, but hitherto, no definitive universal standard exists for the treatment of infantile spasms. Our study provides a developing world perspective on the treatment of infantile spasms with particular reference to two drugs: Vigabatrin and ACTH. First line therapy consisted of either ACTH or Vigabatrin in this study. A second drug was started if the patients showed partial or no response to one of the first line agents. At any given time in the study, patients were put on not more than one drug.

### Birth asphyxia

Cerebral palsy secondary to birth asphyxia/hypoxic ischemic encephalopathy was identified as the leading cause of infantile spasms in our study, contributing to almost 70% of the symptomatic cases. The diagnosis was primarily based on history, neurological examination and brain imaging. The spectrum of underlying etiologies in our study was quite similar to other studies. In a large study, one-third of symptomatic patients had birth related etiology [10]. In another study, hypoxic ischemic encephalopathy was the commonest underlying etiology [11]. According to data from the World Health Organization, 4 - 9 million babies develop birth asphyxia per year and of these, more than 1 million die and roughly the same number develop neurological sequelae [12]. Data from Pakistan has shown that it is one of the leading causes for admission in a neonatal unit [13]. An analysis of neonatal mortality and morbidity in Pakistan has shown that most causes of neonatal morbidity in Pakistan are preventable [14]. In a study conducted in Pakistan, it was reported that lack of antenatal care in mothers, delivery by unskilled birth attendants either at home or a private health facility and prolonged labour were significant risk factors for asphyxial insult at birth [15,16]. Similarly, another study from Pakistan has reported the association between lack of antenatal care, poor nutritional status of mother and antepartum hemorrhage with higher incidence of birth asphyxia [17]. Birth asphyxia may thus be a reflection of the unhealthy baseline maternal status as well as the poor perinatal services offered in maternity homes, communities or hospitals [18]. In order to ameliorate the situation, increased coverage of the population by high quality health services and skilled birth attendants is imperative [19,20]. Evidence based and cost-effective resuscitation strategies for neonates should be implemented [21]. Improvements in proximal risk factors such as female nutrition and prompt treatment as well as prevention of infections should be the long term goals of any policy geared towards improving maternal and child health [17,21]. Similarly, improvement in maternal education should be made a priority in both urban and rural settings.

### Therapeutic parameters

No statistically significant difference was observed between the response rates (based on clinical observation and resolution on EEG when available) of patients put on either ACTH or Vigabatrin therapy initially. Responses to both the first and the second drug were also almost similar, as obvious from the p-values derived from chi-square or fisher's exact testing. However, taking the final outcome at the time of last follow-up into account, it was seen that overall employment of monotherapy prognosticated better remission rates as compared to patients who received a second drug as add-on therapy. It was observed that the majority of the patients who responded to treatment did so within the first few weeks of being put on therapy. However, a full 6 weeks of therapy was allowed in our study before switching to the second drug.

The United Kingdom infantile spasms study comparing vigabatrin with prednisolone or tetracosactide at 14 days was a multicentred randomized controlled trial where the primary outcome was cessation of spasms at day 13 and 14 [22]. According to the study, there was more likelihood of cessation of spasms at day 14 in infants given hormonal treatment than those given Vigabatrin. We didn't observe similar results in our study. Our period of treatment extended well beyond the 14 days parameter used in this study.

Even though younger age at onset has been associated with a worse prognosis [9], but the results in our study were inconsistent with this finding, where younger age at diagnosis appeared to portend better chances of resolution of infantile spasms. We believe that it is possible that earlier diagnosis at a younger age led to earlier administration of therapy which may have led to improved outcomes.

When compared in terms of complete cessation of seizures, both ACTH and Vigabatrin monotherapy as well as add-on therapy showed average cessation rates ranging between 20% and 50%. The results for Vigabatrin are consistent with a class I randomized placebo-controlled trial, which reported 35% response rate [23]. In another large study, 21% to 44% patients responded to Vigabatrin therapy [24]. Our results for ACTH are not in agreement with a meta-analysis that showed a cessation rate ranging from 54% to 100% [25]. Several other studies also report similar results [26,27]. One reason for this inconsistency could be that the aforestated studies have used higher starting doses for ACTH, with 75 IU being the most common dose, as opposed to our study, where the starting dose for 40 IU in almost all cases. This can be further substantiated by two studies that report that ACTH was effective at higher doses, based both on response rate and plasma hormone data [6,28-30].

Overall prognosis in our study was poor. After the onset of spasms, 52/56 (92.8%) children showed developmental delay while only 4 children (7.2%) showed normal developmental milestones. Most of the reports from other countries also report poor outcome in infantile spasms [27].

Considering the final seizure free outcome, etiology and treatment used, it was seen that idiopathic group and symptomatic groups responded well to Vigabatrin as compared to ACTH. These results are consistent with earlier performed studies. In one retrospective study, it was seen that symptomatic children with developmental brain lesions responded at a rate of 83% to vigabatrin versus 63% for ACTH. Cryptogenic spasms responded at a similar rate to both drugs as was seen in our study [31].

With regards to the side-effect profile, Vigabatrin was better than ACTH; with only two patients experiencing the adverse effects, as opposed to ACTH, where six patients reported ACTH-induced hypertension. A previous study has reported arterial hypertension in 11 out of 162 children (6.8%) on ACTH therapy. Mortality in this study has been stated as 4.9%. The other mentioned side effects included infections (being the most common), followed by marked electrolyte disturbances (10 patients), osteoporosis (2 patients), and hypokalaemic alkalosis (2 patients) [32]. Septicemia was the cause of death in both of our patients on ACTH therapy as mentioned above. Vigevano and Cilio have reported hormonal treatments to have greater adverse effects and thus a higher potential to induce mortality [23].

However, in our study, detailed visual field examination could not be carried out in every child receiving Vigabatrin because of financial constraints. This may have lead to underestimation of the side effects of Vigabatrin. Recent reports have established that eye changes occur in patients treated with vigabatrin. A prospective study reports that most children with epileptic syndromes on vigabatrin cannot complain of their eye problems, hence 3-6 monthly ophthalmic follow up is strongly advised, along with regular electroretinography, electro-oculography, and visual evoked potentials if possible [33]. A study from Oman reports one death in a patient on vigabatrin therapy due to aspiration pneumonia [34]. Currently, the benefits of treating infantile spasms with vigabatrin monotherapy seem to outweigh the risks, but further prospective studies and follow-up of children receiving treatment are needed to evaluate the place of vigabatrin in this indication [35]. It is concluded that side effects, even severe ones, are more common during treatment than had been assumed. Careful watch is important before and after treatment. The benefit of very high dosages should also be reconsidered [32]. The side effect profile of vigabatrin dictates that children be started on therapy with a concomitant short admission to the hospital for better observation and institution of aspiration precautions. This, however, has important implications for a developing country like Pakistan where scarce resources are expended on child health care.

Four patients in our study developed Lennox Gastaut Syndrome (LGS) while on therapy. All of these patients had received Vigabatrin initially and had then been put on ACTH. A study from Finland reports 27% of patient with infantile spasms eventually evolving to LGS [36,37]. A review article reported that 20% of all patients with LGS have prior infantile spasms with hypsarrythmia [38]. LGS seizures are often treatment resistant and the long term prognosis is poor [28]. However, we did not come across any study that reports the association of children with infantile spasms on a particular therapy subsequently evolving to LGS.

During 2006 - 2008, we had treated two patients of infantile spasms with prednisolone at a dose of 1.5 mg/kg/day. Although this dose of prednisolone was lower than that used in UKISS study (4 mg/kg/day), it resulted in resolution of spasms. While the demonstration of this aspect with such small numbers is insufficient for analysis or derivation of conclusions, it may be studied further in the future for better validation.

### Strengths and limitations

Literature on pediatric neurology is generally under-represented from the developing countries. In particular, studies on experience with ACTH and Vigabatrin in the treatment of infantile spasms are lacking. Therefore, this study is an important perspective on the management of infantile spasms in the setting of a developing country. To the best of our knowledge, this is the first study of its kind in Pakistan to compare Vigabatrin and ACTH as treatment options for infantile spasms.

However, we acknowledge the following limitations of our study. Conclusions derived from it should be interpreted with these limitations in mind. The study was based on review of patient charts. Our source of data was restricted to hospital records which in turn were reflecting an objective assessment by the physician as well as reliance on parental descriptions for accounts of spasms and seizures. These may not be bias free or accurately recalled in every instance. Secondly, the election of treatment was based on several factors such as availability, cost and ease of administration. The groups were, however, not randomized. As a result, twice as many patients received Vigabatrin as compared to ACTH. All patients didn't get serial EEGs done. In particular, control EEGs were not available for all patients during follow-up. We had divided the groups into symptomatic, cryptogenic and idiopathic. Patients who were suspected of being symptomatic but for whom an underlying structural or biochemical cause could not be identified were classified as cryptogenic. Although patients were evaluated clinically and radiologically while such categorization was done, MRI scans were not performed in all patients due to financial constraints and CT scans were used in some patients as a less expensive alternative. MRI studies are important for defining the cryptogenic groups further.

## Conclusion

Vigabatrin was found to be more efficacious in the treatment of the symptomatic and the idiopathic type of infantile spasm. However, Vigabatrin and ACTH showed no significant difference in the initial treatment of infantile spasms. These results are consistent with the findings stated in the practice parameter published in 2004 in Neurology by the American academy of Neurology (AAN) and Child Neurology Society that bases its recommendations for the treatment of infantile spasms on two large surveys conducted in the US and Japan [25]. Patients receiving ACTH were 1.2 times more likely to relapse as compared to the patients receiving Vigabatrin when considering monotherapy. Younger age at diagnosis of infantile spasms portends resolution of seizures as compared to older age. Larger studies are required to validate the therapeutic trends observed in our study. Birth asphyxia, a potentially preventable condition, was a leading etiology of symptomatic infantile spasms in our study. We need to gear efforts towards reducing the incidence of birth asphyxia in our part of the world through carefully tailored strategies. This will have an enormous impact by reducing a preventable cause of symptomatic infantile spasms. Despite all the advances in understanding the various treatment options available, infantile spasms still remains an elusive childhood disease that is difficult to treat with the overall prognosis being dismal. The search for an ideal drug therefore continues - a drug that is not only effective in controlling spasms, has minimal side effects and improves long term outcome, but is also easily accessible to most patients [6].

## Competing interests

The authors declare that they have no competing interests.

## Authors' contributions

SG, SI and TS collected, entered and analyzed the data and wrote the manuscript. SI was involved in study conception and also supervised the project in addition to writing and editing the manuscript. All authors read and approved the final manuscript.

## Pre-publication history

The pre-publication history for this paper can be accessed here:

http://www.biomedcentral.com/1471-2431/10/1/prepub
